# The Role of Death-Anxiety-Induced Fear of COVID-19 in Compliance With and Acceptance of Government-Issued COVID-19 Regulations

**DOI:** 10.3389/fpsyg.2022.881603

**Published:** 2022-05-02

**Authors:** Hugo M. Kehr, Cafer Bakaç, Marius Jais, Dorothee Brunner, Julian Voigt, Lea Holzemer

**Affiliations:** Technical University of Munich, Munich, Germany

**Keywords:** death anxiety, fear of COVID-19, terror management theory, compliance with COVID-19 regulations, acceptance of COVID-19 regulations

## Abstract

The present research was conducted to empirically examine whether death anxiety is the fundamental fear that feeds people’s fear of COVID-19 and leads to increased behavioral compliance with and acceptance of COVID-19 regulations. Results from an online survey of 313 participants from New York City show that death anxiety was, indeed, positively associated with behavioral compliance with, but not acceptance of, COVID-19 regulations *via* an increased fear of COVID-19. Hence, media campaigns that are designed to increase people’s compliance with restrictive COVID-19 measures by stirring up their death anxiety are likely to meet their target, but they do not necessarily lead to increased public acceptance of the measures taken.

## Introduction

Since its outbreak in late 2019, the COVID-19 pandemic has had a devastating impact on global health and the world economy, with an enormous death toll and economic losses exceeding hundreds of billions of United States Dollars (Nicola et al., 2020; Weiss et al., 2021). In attempting to restrain the spread of COVID-19, governments around the world reacted by imposing severe restrictions on their people, some of which (e.g., lockdowns and school closures) violated fundamental human rights (Quintana and Uriburu, 2020; Thomson and Ip, 2020). Supported by the press, government officials explained and defended their restrictive measures by stressing the deadly potential of COVID-19, with TV channels perpetuating footage of overcrowded emergency rooms, dead bodies, and piled up coffins (Reed, 2020). Public health campaigns were crafted to raise the salience of death, which researchers have argued may have led to an increase in people’s death anxiety (Menzies and Menzies, 2020; Pyszczynski et al., 2021). Clearly, the notion underlying these campaigns was that, by heightening the salience of personal vulnerability and death, fear of COVID-19 would increase, and people would be more likely to comply with and accept the severe and unprecedented restrictions. However, this proposition has yet to be empirically tested. Therefore, the present research was conducted to empirically test whether death anxiety is, indeed, positively associated with fear of COVID-19 and, subsequently, with behavioral compliance with and acceptance of COVID-19 regulations.

### Death Anxiety and Fear of COVID-19

Death anxiety, the fear of the threat of non-being, has been proposed to be a central and universal part of human existence (Becker, 1993). Researchers have identified death anxiety as a basic and fundamental fear that lies at the root of numerous psychological conditions (e.g., hypochondriasis, panic disorder, and anxiety disorders) and have therefore suggested that it should be considered a transdiagnostic construct (Iverach et al., 2014). Accordingly, death anxiety can fuel other, more specific fears (e.g., fear of illnesses). In line with this, researchers recently proposed that death anxiety may also predict fear of COVID-19 (Menzies and Menzies, 2020).

According to Terror Management Theory (TMT), there are two different defense mechanisms against death anxiety, depending on whether death anxiety lies within or outside of a person’s conscious awareness: proximal and distal defenses (Pyszczynski et al., 2015, 2021; Menzies and Menzies, 2020). When people consciously experience death anxiety, they employ proximal defenses to alleviate the fear (Pyszczynski et al., 2015). Proximal defenses include suppressing death-related thoughts (e.g., by avoiding social media) or trying to prevent death (e.g., by staying home; Iverach et al., 2014; Menzies and Menzies, 2020). Conversely, distal defenses (e.g., enhancing one’s self-esteem or endorsing one’s worldview) are employed when people subconsciously experience death anxiety (Menzies and Menzies, 2020; Pyszczynski et al., 2021). Because COVID-19-related measures (e.g., social distancing, heightened hygiene, or avoidance of public transportation) are proximal defenses, the present research focuses on proximal defenses and consciously experienced death anxiety. We propose that people who consciously experience death anxiety will be fearful of COVID-19 and will subsequently adhere to government-imposed COVID-19 regulations.

There is evidence from recent research that fear of COVID-19 is indeed associated with increased adherence to government-imposed COVID-19-related measures. Harper et al. (2021) found that fear of COVID-19 was associated with heightened behavioral compliance with government-mandated personal restrictions, and Zettler et al. (2020) reported that heightened levels of anxiety and fear were associated with a greater level of acceptance of COVID-19-related measures.

Interestingly, most studies on adherence to COVID-19 regulations have not differentiated between compliance with and acceptance of COVID-19 regulations (e.g., Sabat et al., 2020) or have focused entirely on compliance rather than acceptance (e.g., Harper et al., 2021; Morbée et al., 2021). The study by Franzen and Wöhner (2021) is a notable exception. In a Swiss sample of young adults, the authors found that individuals living with a member of a risk group had higher compliance with — but not higher acceptance of—COVID-19 measures. The authors also reported that individuals’ perceptions of the social risk of the coronavirus led to increased acceptance of the COVID-19 measures, but their perceptions of personal risk did not.

In sum, death anxiety has been identified as a potential source of increased fear of COVID-19 (Menzies and Menzies, 2020), and fear of COVID-19 has been shown to be associated with increased compliance with and acceptance of government-imposed COVID-19 restrictions (Zettler et al., 2020; Franzen and Wöhner, 2021; Harper et al., 2021). Further, consciously experienced death anxiety is known to entail proximal defenses (Pyszczynski et al., 2015), presumably including compliance with and acceptance of COVID-19 restrictions. From the above, our prediction was that death anxiety drives fear of COVID-19, which subsequently leads to increased compliance with and acceptance of COVID-19 restrictions. Because no studies have examined relationships between death anxiety and compliance with and acceptance of COVID-19 restrictions or identified fear of COVID-19 as a potential mediator of this relationship, we conducted this empirical study.

### Hypotheses

From the above, we derived the following hypotheses^1^ :

H1: Death anxiety is significantly positively associated with fear of COVID-19.

H2a: Death anxiety is significantly positively associated with behavioral compliance with COVID-19 regulations.

H2b: Death anxiety is significantly positively associated with acceptance of COVID-19 regulations.

H3a: Fear of COVID-19 is significantly positively associated with behavioral compliance with COVID-19 regulations.

H3b: Fear of COVID-19 is significantly positively associated with acceptance of COVID-19 regulations.

H4a: The positive effect of death anxiety on behavioral compliance with COVID-19 regulations is mediated by fear of COVID-19.

H4b: The positive effect of death anxiety on acceptance of COVID-19 regulations is mediated by fear of COVID-19.

In view of Open Science recommendations, all materials concerning the study, analyses, and data are publicly available on the Open Science Framework at https://osf.io/fvmjr/?view_only= 8c55d0ebeeca4798a8636a572e834d30. Furthermore, we preregistered our hypotheses, study design, sampling plan, variables, and analysis plan on the Open Science Framework publicly available at https://osf.io/sx5kv?view_only=7b1b3260 cffc47f5ba22329b02e9e003. There are only minor deviations from our preregistration, which we report in the following sections.

## Materials and Methods

### Data Collection Procedure and Participants

With an expected small indirect effect (*ab* = 0.04; α = 0.05, β = 0.80), our statistical power analysis (Kenny, 2017) yielded a sample size requirement of 252 participants. However, we decided to aim for a sample size of 400 participants (a) to allow for expected attrition, (b) to further increase our statistical power to detect even very small effects, and (c) because the sample size would be comparable to other recent research on fear of COVID-19 (Mertens et al., 2020).

We collected data exclusively from New York City citizens because New York City was heavily affected by the COVID-19 outbreak (Armstrong et al., 2020), which led to a number of government regulations. At the same time, this limited sampling approach allowed us to ensure comparability of participants by ruling out local differences in government regulations to prevent the spread of the virus. We recruited participants using Amazon’s Mechanical Turk (MTurk), which has been shown to be as reliable as traditional data collection methods for social research (Buhrmester et al., 2011). After a predetermined 20-day collection period that began on March 13, 2021, data collection was stopped to prevent uncontrolled systematic variability in light of the rapidly changing pandemic situation, including government regulations. We finally reached a sample size of 313 participants from New York City (*M*_age_ = 37.65, 52.72% women), thereby meeting the sample size requirement calculated in our power analysis. In line with our preregistration, we excluded two participants on the basis of Mahalanobis distance. Note that the results reported below remained the same even when outliers were included in the analyses.

### Online Questionnaire and Materials

Data were collected with an online questionnaire on SoSci Survey. The study met relevant ethical guidelines, including adherence to the local legal requirements. After participants gave their informed consent to the terms and conditions of the study, they were presented with the questionnaires. The survey questions followed the same sequence as listed below. At the end of the survey, we asked for biographical data (age, gender, education, profession, and income).

#### Death Anxiety (Independent Variable)

We measured death anxiety with Templer’s Death Anxiety Scale (Templer, 1970), a “reliable and valid measure of death anxiety” (Iverach et al., 2014, p. 584) in its original 15-item version, according to Zuccala et al. (2019), “the most widely used measure” (p. 2) of death anxiety. Participants were asked to indicate the extent to which they agreed or disagreed with each statement on a 5-point Likert scale ranging from 1 (*strongly disagree*) to 5 (*strongly agree*). A sample statement is, “I often think about how short life really is” (α = 0.86, *M* = 3.27, *SD* = 0.68).

#### Fear of COVID-19 (Mediator)

We assessed fear of COVID-19 using a recently developed and validated seven-item measure, namely, the Fear of COVID-19 Scale (Ahorsu et al., 2020). Participants were asked to indicate the extent to which they agreed or disagreed with each statement using a 5-point Likert scale ranging from 1 (*strongly disagree*) to 5 (*strongly agree*). A sample statement is, “I cannot sleep because I’m worrying about getting coronavirus-19” (α = 0.93, *M* = 2.61, *SD* = 1.06).

#### Behavioral Compliance With COVID-19 Regulations (Dependent Variable)

We measured participants’ behavioral compliance with COVID-19 regulations using a scale we developed in close reference to Roma et al.’s (2020) behavioral compliance scale. Six items were extracted from the ‘‘Social Distancing and Face Covering Rules’’ issued in March 2021 by the City of New York^2^ that were, at that time, identical to the precautions recommended by the ‘‘Centers for Disease Control and Prevention’’^3^ (CDC). Participants were asked, “Please indicate how often you have shown the recommended behavior during the last 3 months.” A sample item is, “I avoided public gatherings of more than 10 people.” Answers were given on a 5-point Likert scale ranging from 1 (*never*) to 5 (*always*). Psychometric properties of the scale were good (α = 0.86, *M* = 4.16, *SD* = 0.82).

#### Acceptance of COVID-19 Regulations (Dependent Variable)

We measured acceptance of COVID-19 regulations analogously to the behavioral compliance with COVID-19 regulations scale, but the wording was altered slightly. Participants were asked, “Now, we would like to know how appropriate you find the suggestions made by the City of New York.” A sample item is, “To avoid public gatherings of more than 10 people.” Answers were given on a 5-point Likert scale that ranged from 1 (*too strict*) to 5 (*not strict enough*). The scale was reverse-coded so that higher scores represented greater acceptance of COVID-19 regulations. Psychometric properties of the six-item scale were good (α = 0.93, *M* = 3.03, *SD* = 0.75).

#### Fear of Losing Loved Ones (Control Variable)

We measured fear of losing loved ones because (a) research has shown that fear of one’s own death should be distinguished from fear of losing significant others (see Cuniah et al., 2021, for a recent publication) and (b) risk for loved ones has been found to be one of the strongest predictors of COVID-19-related fears (Mertens et al., 2020). Thus, we used the eight-item subscale The Death of Others from The Collett-Lester’s Fear of Death Scale Version 3.0 (Lester, 1990; Lester and Abdel-Khalek, 2003) to control for potential influences on our focal variables. Participants were asked to indicate “how disturbed or anxious” they felt after reading statements about the deaths of others on a 5-point Likert scale ranging from 1 (*not*) to 5 (*very*). A sample item is, “Losing someone close to you” (α = 0.86, *M* = 3.50, *SD* = 0.94).

#### Financially Affected by COVID-19 (Control Variable)

Because the financial situation of many individuals has gotten worse during the pandemic (Horowitz et al., 2021), which is likely to affect COVID-19-related perceptions and behaviors, we used a single self-designed item and asked participants to indicate “How did the pandemic affect your financial situation?” on a 5-point Likert scale ranging from 1 (*worsened strongly*) to 5 (*improved strongly*) (*M* = 3.27, *SD* = 0.90). We reverse-coded this variable so that higher values indicate a greater financial impact of COVID-19.

#### Professionally Affected by COVID-19 (Control Variable)

Because many individuals’ job situations have gotten worse during the pandemic (Horowitz et al., 2021), which is likely to affect COVID-19-related perceptions and behaviors, we used a single self-designed item and asked participants to rate “How did the pandemic affect your job opportunities?” on a 5-point Likert scale ranging from 1 (*worsened strongly*) to 5 (*improved strongly*) (*M* = 3.36, *SD* = 0.98). We reverse-coded this variable so that higher values indicate a greater financial impact of COVID-19.

#### Media Exposure (Control Variable)

Because media exposure related to the COVID-19 outbreak has been found to be associated with an increased fear of COVID-19 (Mertens et al., 2020), we used a single-item scale that has been applied in previous research (Mertens et al., 2020) and asked participants, “Have you looked up any extra information regarding the COVID-19 outbreak? (not taking into account coincidentally seeing/reading about it in the news)” using a *Yes* or *No* answer format (*M* = 0.71, *SD* = 0.45).

#### Political Orientation (Control Variable)

Because conservatism has been shown to be associated with less perceived personal vulnerability to COVID-19 and less perceived severeness of the pandemic (Calvillo et al., 2020), we assessed political orientation using the scale by Kanai et al. (2011)^4^. Participants were asked to indicate their political orientation on a single-item scale with the levels: *very conservative* (1), *conservative* (2), *middle-of-the-road* (3), *liberal* (4), and *very liberal* (5) (α = 0.93, *M* = 3.36, *SD* = 1.07).

#### Relationship to Conspiracy Theories (Control Variable)

Because COVID-19-related conspiracy theories were associated with resistance to preventive behaviors (Romer and Jamieson, 2020), we used the single-item scale by Lantian et al. (2016) to assess participants’ relationship to conspiracy theories. Participants were asked to indicate whether the item, “I think that the official version of the events given by the authorities very often hides the truth” was true or false on a 9-point Likert scale ranging from 1 (*completely false*) to 9 (*completely true*), *M* = 5.47, *SD* = 2.00.

#### General Health (Control Variable)

Because bad general health is related to higher mortality from COVID-19 (e.g., Zhou et al., 2020), high-risk individuals may perceive a greater personal threat in the COVID-19 pandemic (Mertens et al., 2020). Thus, we measured general health by using a single-item scale that has been applied in previous research (Mertens et al., 2020). Participants were asked to answer the item “Overall, I would rate my general health as” on a 5-point Likert scale ranging from 1 (*extremely bad*) to 5 (*extremely good*), *M* = 3.89, *SD* = 0.82.

#### Perceived Disease Vulnerability (Control Variable)

We measured perceived disease vulnerability for two reasons: First, because greater perceived vulnerability to disease was found to be related to a greater belief that public health measures protect the population (De Coninck et al., 2020), and second, and more importantly, because heightened perceived disease vulnerability has been associated with increased virus anxiety (Jungmann and Witthöft, 2020). We used the 15-item Perceived Vulnerability to Disease Scale (Duncan et al., 2009). Participants indicated the extent to which they believed they were vulnerable to diseases on a 7-point Likert scale ranging from 1 (*strongly disagree*) to 7 (*strongly agree*). A sample item is, “In general, I am very susceptible to colds, flu, and other infectious diseases” (α = 0.72, *M* = 4.38, *SD* = 0.80).

#### Statistical Analyses

In line with and Preacher and Hayes (2004, 2008) we conducted two mediation analyses to test our main mediation hypotheses that fear of COVID-19 mediates the effect of death anxiety on behavioral compliance with and acceptance of COVID-19 regulations. For the analyses, we entered death anxiety as the predictor, fear of COVID-19 as the mediator, and behavioral compliance with and acceptance of COVID-19 regulations as outcome variables. Additionally, we added the variables that were specified as control variables to our mediational models as covariates to statistically control for their effects on the dependent variables. We computed 95% confidence intervals from 10,000 bootstrapping resamples for each indirect effect to test for whether the indirect effects of the focal predictor on the outcome variables through the mediator were significantly different from zero (Shrout and Bolger, 2002). We used the IBM SPSS (Version 28) PROCESS macro (Model 4; Hayes, 2018) for all the analyses. Using the same statistical procedure, we also tested all of our hypotheses without the control variables.

Additionally, we estimated equivalent models to test the alternative possibility that death anxiety mediates the effect of fear of COVID-19 on behavioral compliance with and acceptance of COVID-19 regulations. In these equivalent models, we reversed the predictor and the mediators, so that fear of COVID-19 became the predictor and death anxiety became the mediator.

## Results

### Descriptive Statistics and Correlations

Descriptive statistics and correlations among study variables can be found in Table 1. We found that only 2% of our sample reported intense death anxiety (i.e., mean plus one standard deviation). Further, we found that women reported more death anxiety than men (correlation between death anxiety and gender; *r* = -0.20, *p* < 0.01). We also found that age, level of education, and income were not significantly correlated with death anxiety (*r* = −0.06, *r* = −0.05, and *r* = −0.09, respectively, all *ps* > 0.05).

**TABLE 1:** Means, standard deviations, and zero-order correlations between all study variables.

Variable	*M*	*SD*	1	2	3	4	5	6	7	8	9	10	11	12	13	14	15
1. Gender*^a^*	0.47	0.50															
2. Age	37.65	11.31	–0.03														
3. Income	7.41	3.02	0.09	0.14*													
4. Education	6.46	1.71	0.02	0.05	0.42**												
5. Death anxiety (IV)	3.27	0.68	−0.20**	–0.06	–0.05	–0.09											
6. Fear COVID-19 (MV)	2.61	1.06	–0.06	−0.13*	−0.14*	0.03	0.55**										
7. Fear loved ones	3.50	0.94	−0.16**	–0.08	–0.09	−0.13*	0.56**	0.29**									
8. PDV	4.38	0.80	–0.04	–0.03	–0.07	0.01	0.28**	0.46**	0.07								
9. Conspiracy	5.47	2.00	–0.01	–0.07	–0.07	–0.08	–0.02	0.06	–0.02	0.04							
10. General health	3.89	0.82	0.05	−0.12*	0.26**	0.17**	−0.16**	–0.10	–0.09	−0.27**	–0.04						
11. PO	3.36	1.07	−0.19**	–0.09	–0.05	0.08	0.10	0.04	0.21**	–0.09	−0.30**	–0.07					
12. Media exposure	0.71	0.45	–0.04	–0.01	–0.07	–0.06	0.05	0.10	0.07	0.04	0.09	–0.03	0.14*				
13. FA	3.27	0.90	–0.09	–0.06	−0.20**	–0.05	0.18**	0.08	0.23**	–0.03	–0.08	−0.12*	0.19**	0.10			
14. PA	3.36	0.98	–0.10	−0.11*	−0.21**	–0.00	0.23**	0.03	0.28**	–0.02	−0.12*	–0.07	0.28**	0.04	0.66**		
15. Compliance (DV)	4.16	0.82	−0.13*	0.11	–0.05	–0.00	0.23**	0.30**	0.29**	0.11	−0.19**	–0.06	0.35**	0.19**	0.23**	0.27**	
16. Acceptance (DV)	2.97	0.75	–0.11	–0.07	–0.09	–0.05	0.23**	0.17**	0.30**	0.04	−0.11*	–0.05	0.44**	0.07	0.24**	0.29**	0.50**

*N = 313. IV, independent variable; MV, mediator variable; DV, dependent variable; Fear loved ones, fear of losing loved ones; PDV, perceived disease vulnerability; Conspiracy, relationship to conspiracy theories; PO, political orientation; FA, financially affected by COVID-19; PA, professionally affected by COVID-19; Compliance, behavioral compliance with COVID-19 regulations; Acceptance, acceptance of COVID-19 regulations.*

^a^Coding for gender: female = 0; male = 1. *p < 0.05; **p < 0.01.

Additionally, we examined the zero-order correlations between predictor, mediator, and outcome variables (see Table 1). We found a significant correlation between death anxiety and fear of COVID-19 (*r* = 0.55, *p* < 0.01). We also found that death anxiety and fear of COVID-19 were both significantly correlated with behavioral compliance with COVID-19 regulations (*r* = 0.23 and *r* = 0.17, respectively, both *ps* < 0.01) and with acceptance of COVID-19 regulations (*r* = 0.23 and *r* = 0.30, respectively, both *ps* < 0.01).

As shown in Table 1, we found that the extents to which individuals were financially and professionally affected by COVID-19 were significantly and positively associated with behavioral compliance with COVID-19 regulations (financial: *r* = 0.23, *p* < 0.01; professional: *r* = 0.27, *p* < 0.01) and with acceptance of COVID-19 regulations (financial: *r* = 0.24 *p* < 0.01; professional: *r* = 0.29 *p* < 0.01).

### Main Hypothesis Testing

**FIGURE 1:**
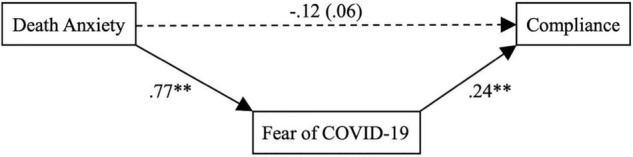
Mediation model for the influence of death anxiety on compliance with COVID-19 regulations through fear of COVID-19. *N* = 313. The value in parentheses represents the direct effect of death anxiety on compliance with COVID-19 regulations without controlling for fear of COVID-19. ^**^p < 0.01.

When statistically controlling for our covariates, we found a significant relationship between death anxiety and fear of COVID-19 [*b* = 0.77, *SE* = 0.08, *t*(311) = 9.14, *p* < 0.001; *R*^2^ = 0.47]. Additionally, we found the expected significant association between fear of COVID-19 and behavioral compliance with COVID-19 regulations [*b* = 0.24, *SE* = 0.05, *t*(311) = 4.74, *p* < 0.001; *R*^2^ = 0.32], but not with acceptance of COVID-19 regulations [*b* = 0.06, *SE* = 0.05, *t*(311) = 1.34, *p* > 0.05; *R^2^* = 0.27]. Further, albeit death anxiety was not significantly associated with behavioral compliance with COVID-19 regulations [*b* = 0.06, *SE* = 0.08, *t*(311) = 0.84, *p* > 0.05; *R*^2^ = 0.26], the predicted indirect effect of death anxiety on behavioral compliance with COVID-19 regulations through fear of COVID-19 was found to be significant (*ab* = 0.19, *SE* = 0.05, and 95% CI [0.10, 0.29]). Figure 1 illustrates the results. However, death anxiety was neither directly [*b* = 0.08, *SE* = 0.07, *t*(311) = 1.17, *p* > 0.05; *R*^2^ = 0.27] nor indirectly (*via* fear of COVID-19) related to acceptance of COVID-19 regulations (*ab* = 0.05, *SE* = 0.05, and 95% CI [−0.04, 0.15]). Additionally, we tested our hypotheses without controlling for the covariates, and found that all aforementioned study results remained largely intact.

We also estimated equivalent models to test the alternative possibility that death anxiety mediated the effects of fear of COVID-19 on behavioral compliance with and acceptance of COVID-19 regulations, respectively. Results showed that although fear of COVID-19 was significantly associated with death anxiety [*b* = 0.29, *SE* = 0.03, *t*(311) = 9.14, *p* < 0.001; *R*^2^ = 0.53], death anxiety did not significantly predict either behavioral compliance with COVID-19 regulations [*b* = 0.12, *SE* = 0.08, *t*(311) = −1.45, *p* > 0.05; *R*^2^ = 0.32] or acceptance of COVID-19 regulations [*b* = 0.03, *SE* = 0.08, *t*(311) = 0.41, *p* > 0.05; *R*^2^ = 0.27]. Consequently, the indirect effects of fear of COVID-19 on behavioral compliance with or acceptance of COVID-19 regulations through death anxiety were non-significant (*ab* = −0.03, *SE* = 0.03, and 95% CI [−0.09, 0.02]; *ab* = 0.01, *SE* = 0.02, 95% CI [−0.03, 0.05], respectively).

## Discussion

The results of this study show that only 2% of the sample showed intense death anxiety which is in line with the literature (Agras et al., 1969). We also found that, in general, the demographic correlates of death anxiety were similar to findings reported in the death anxiety literature (Kastenbaum, 2000; Furer et al., 2007). The examination of the relationships of the covariates with the outcome variables revealed that the extents to which individuals were financially and professionally affected by COVID-19 were positively associated with their behavioral compliance with and acceptance of COVID-19 regulations, findings that support the previous literature (e.g., Han et al., 2021).

With respect to our hypotheses, the results show that death anxiety was significantly associated with fear of COVID-19, thus corroborating Hypothesis 1. In addition, with respect to compliance with COVID-19 regulations, the results were largely in line with predictions. Specifically, fear of COVID-19 was significantly associated with compliance with COVID-19 regulations, corroborating Hypothesis 3a. Further, and most importantly, even though we did not find the expected direct link between death anxiety and compliance with COVID-19 regulations, as predicted in Hypothesis 2a, we did find the expected indirect effect of death anxiety on compliance with COVID-19 regulations via fear of COVID-19, corroborating Hypothesis 4a, the mediator hypothesis. Conversely, with respect to acceptance of COVID-19 regulations, the results were not in line with predictions. Specifically, we did not find the expected positive relationships between death anxiety or fear of COVID-19 and acceptance of COVID-19 regulations (Hypotheses 2a and 3a). Consequently, there was no significant indirect effect of death anxiety on acceptance of COVID-19 regulations *via* fear of COVID-19, and, therefore, no support for Hypothesis 4b.

In sum, this study corroborates the notion that death anxiety is the fundamental fear (Iverach et al., 2014) that underlies the fear of COVID-19, in line with psychological researchers’ speculations (Menzies and Menzies, 2020). In turn, fear of COVID-19 drives behavioral compliance with government regulations to avert the spread of COVID-19. The results were obtained after statistically controlling for several prominent covariates. Notably, controlling for the influences of perceived disease vulnerability (Jungmann and Witthöft, 2020) and fear of losing loved ones (Mertens et al., 2020; Cuniah et al., 2021) allowed us to examine and highlight the unique effects of death anxiety on our dependent variables. Nevertheless, it is important to note that all study results remained intact when the covariates were not statistically controlled for. Further, our results did not speak in favor of the alternative possibility that death anxiety mediates the effect of fear of COVID-19 on behavioral compliance with and acceptance of COVID-19 regulations.

Without overinterpreting our inability to reject the null hypothesis, our findings seem to indicate that behavioral compliance with and acceptance of COVID-19 regulations need to be differentiated. In fear of COVID-19, people may behaviorally comply with restrictive measures imposed by their governments, but they do not necessarily accept them. Hence, media campaigns that are designed to increase people’s compliance with restrictive COVID-19 measures by stirring up their death anxiety (Mertens et al., 2020; Pyszczynski et al., 2021) are likely to meet their target, but they will not necessarily lead to increased public acceptance of the measures taken. In addition, researchers have cautioned that social media exposure that leads to persistent salience of the threat of COVID-19 to mortality may be associated with poorer mental health (Iverach et al., 2014; Mertens et al., 2020; Pyszczynski et al., 2021).

Clearly, this study is not without limitations. First, it is a cross-sectional study. Therefore, it is not possible to infer causality from the findings. Indeed, an alternative view on the underlying causality could be that footage portraying dead victims from COVID-19 may have directly triggered fear of COVID-19 as an antecedent of subsequently increased death anxiety. To us, the alternative causality chain seems rather unlikely. First, our empirical findings indicate that there was no significant indirect path from fear of COVID-19 to behavioral compliance *via* death anxiety. Second, and more importantly, death anxiety theorists have long held that death anxiety should be conceptualized as a fundamental and integral part of human existence (Becker, 1993) and that it can be the underlying cause of more specific fears of certain illnesses (Iverach et al., 2014), including fear of COVID-19 (Menzies and Menzies, 2020). In and of itself, a new virus is not necessarily threatening, but footage portraying death as the paradigmatic example of consequences of the COVID-19 pandemic may trigger death anxiety and subsequently spill over to instigate fear of COVID-19. But clearly, additional laboratory studies that manipulate death anxiety and subsequently assess behavioral compliance with COVID-19 regulations are essential in the future. Further, the survey was conducted online with participants from New York City so that results may have been affected by some respondent bias. Finally, although many covariates were controlled for, the possibility of unmeasured factors causing some residual confounding cannot be excluded.

## Data Availability Statement

The datasets presented in this study can be found in online repositories. The names of the repository/repositories and accession number(s) can be found below: https://osf.io/fvmjr/?view_only=8c55d0ebeeca4798a8636a572e834d30.

## Ethics Statement

Ethical review and approval was not required for the study on human participants in accordance with the Local Legislation and Institutional Requirements. The patients/participants provided their written informed consent to participate in this study.

## Author Contributions

CB performed the data collection and analysis. HK, CB, and MJ wrote the first draft of the manuscript. All authors contributed to the study conception, design, and material preparation, commented on previous versions of the manuscript, and read and approved the final manuscript.

## Conflict of Interest

The authors declare that the research was conducted in the absence of any commercial or financial relationships that could be construed as a potential conflict of interest.

## Publisher’s Note

All claims expressed in this article are solely those of the authors and do not necessarily represent those of their affiliated organizations, or those of the publisher, the editors and the reviewers. Any product that may be evaluated in this article, or claim that may be made by its manufacturer, is not guaranteed or endorsed by the publisher.

## Footnotes

1 These hypotheses differ slightly from the preregistered hypotheses. Specifically, our preregistration contained only three hypotheses. Each of these comprised two separable dependent variables: behavioral compliance with COVID-19 regulations and acceptance of COVID-19 regulations. For this article, however, we decided it would be clearer if we formulated these three hypotheses for each of the dependent variables separately. Also, the preregistration did not specify H1, the hypothesis that death anxiety is significantly associated with fear of COVID-19, because this prediction was included in the mediation hypotheses (H4a and H4b). However, for this article, we decided to be more specific and spell out H1. Further, in our preregistration, we used “fear of death” instead of “death anxiety” for our hypotheses. In fact, in our preregistration, we employed “death anxiety” and “fear of death” interchangeably, as other researchers on death anxiety have done (e.g., Menzies and Menzies, 2020). For this article, however, we decided to use “death anxiety” throughout to be consistent. Also, we changed the labeling of “COVID-19 restrictions” to “COVID-19 regulations” because this term seems more neutral. Lastly, we added the directions of the hypothesized associations. We must clarify that we did not add or delete any content from the preregistered hypotheses, so the new hypotheses are essentially identical to the preregistered ones.

2 Retrieved from: https://portal.311.nyc.gov/article/?kanumber=KA-03297.

3 Retrieved from: https://www.cdc.gov/coronavirus/2019-ncov/prevent-getting-sick/prevention.html.

4 Note that our preregistration included three scales for political orientation, but we used only the one specified above in our analyses. We did so because the political orientation scale specified above had better psychometric properties than the others, and all study results remained basically the same with and without the other scales included in the analyses.
